# Supplementary material for: Mycobacterium abscessus Opsonization Allows an Escape from the Defensin Bactericidal Action in Drosophila
Source: Microbiol Spectr. 2023 Jun 1;11(4):e00777-23. doi: 10.1128/spectrum.00777-23 (PMC10434004; doi:10.1128/spectrum.00777-23)
Supplement: Supplemental file 1 — Supplemental material. Download spectrum.00777-23-s0001.pdf, PDF file, 0.2 MB [file spectrum.00777-23-s0001.pdf]

Figure S1

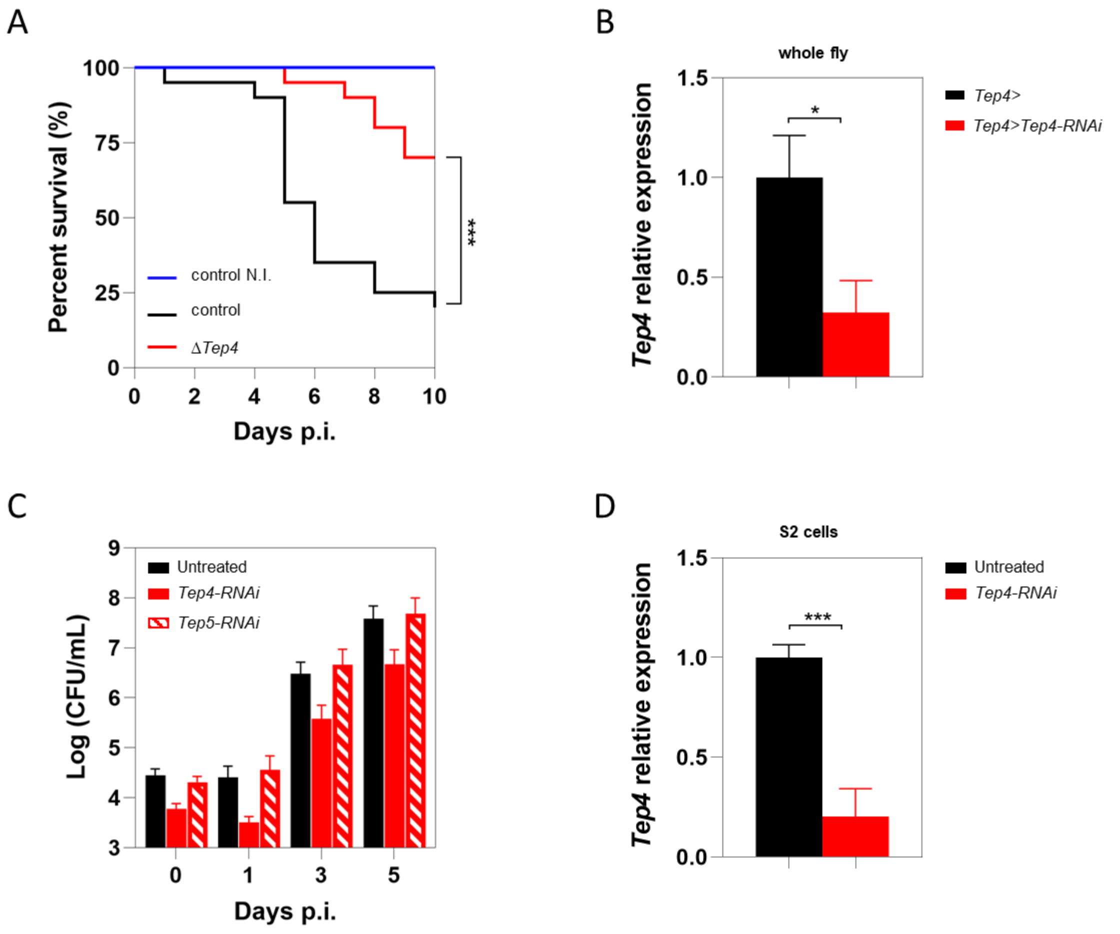

Figure S1. Tep4 deficient flies survive better to *M. abscessus* infection.

(A-D) (A) Survival curves of water injected w1118 (control N.I.) or w1118 (control) and Tep4 mutant (ΔTep4) flies injected with 10 CFU of *M. abscessus*.

(B) Quantification of *Tep4* gene relative expression by qRT-PCR. RNA was extracted on 5-7 days old female *Tep4>* and *Tep4>Tep4-RNAi* flies. Plots represent the normalized expression according to *Tep4>*.

(C) Intracellular growth of *M. abscessus* in untreated, Tep4 or Tep5 specific dsRNA-treated S2 cells. Bacterial load was quantified by CFU counting on days 0, 1, 3 and 5.

(D) Quantification of *Tep4* gene relative expression by qRT-PCR. RNA was extracted on untreated and Tep4-RNAi treated cells. Plots represent the normalized expression according to untreated cells. Survivals were analyzed on 40-60 flies per condition using log-rank test and the relative expressions by student *t-test* (*p<0.05; ***p<0.001).

Figure S2

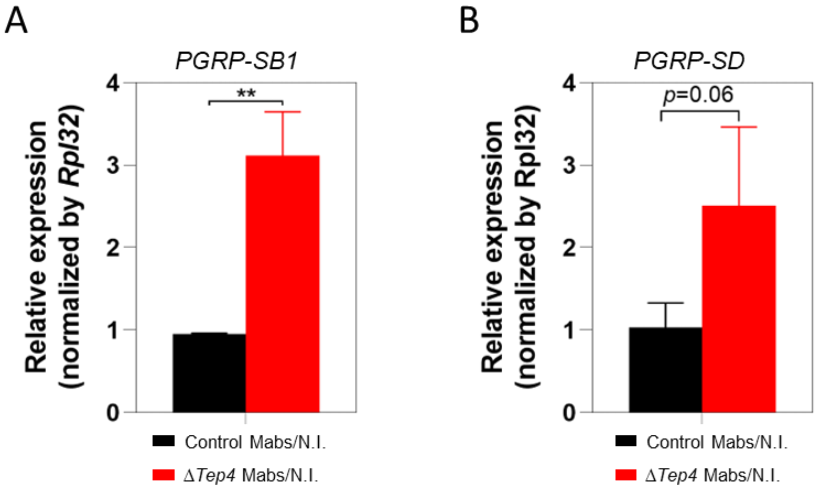

Figure S2. *PGRP-SB1* and *PGRP-SD* are upregulated in *M. abscessus*-infected *Tep4* mutant flies. (A-B) Quantification of *PGRP-SB1* (A) and *PGRP-SD* (B) genes relative expression by qRT-PCR. RNA was extracted on day 3 post-infection from w1118 and ΔTep4 flies injected with water or 10 CFU (Colony Forming Unit) of *M. abscessus*. Plots represent the ratio of expression level in infected on non-infected flies.Relative expressions were compared using student *t-test* (*p<0.05).

Figure S3

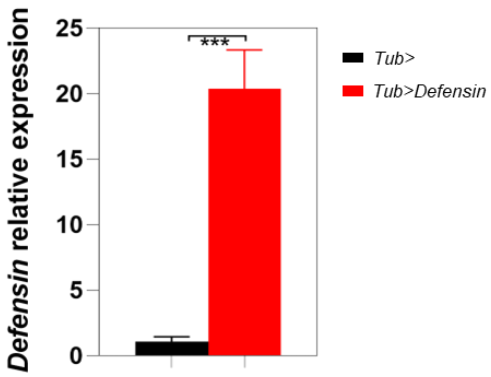

Figure S3. Validation of the transgene allowing *Defensin* overexpression. Quantification of *Defensin* gene relative expression by qRT-PCR. RNA was extracted on 5-7 days old female *Tub>* and *Tub>Defensin* flies. Plots represent the normalized expression according to *Tub>*. Relative expressions were compared using student *t-test* (***$p<0.001$)
